# Immune Modulation During Treatment with Enzalutamide Alone or with Radium-223 in Patients with Castration Resistant Prostate Cancer

**DOI:** 10.3390/cancers17101730

**Published:** 2025-05-21

**Authors:** Peter D. Zang, Diane M. Da Silva, Zhang-Xu Liu, Shivani Kandukuri, Denice Tsao-Wei, Anishka D’Souza, W. Martin Kast, Sumanta K. Pal, Cheryl Kefauver, Maribel Juanqueira, Lixin Yang, David I. Quinn, Tanya B. Dorff

**Affiliations:** 1 Department of Medical Oncology and Therapeutics Research, City of Hope, 1500 East Duarte Road, Duarte, CA 91010, USA; 2 Department of Obstetrics & Gynecology, USC/Norris Comprehensive Cancer Center, 1450 Biggy Street, Los Angeles, CA 90033, USA; 3 Beckman Center for Immune Monitoring, USC/Norris Comprehensive Cancer Center, 1450 Biggy Street, Los Angeles, CA 90033, USA; 4 Clinical Pathology, USC/Norris Comprehensive Cancer Center, 1500 San Pablo Street, Los Angeles, CA 90033, USA; 5 Department of Preventative Medicine, USC/Norris Comprehensive Cancer Center, 1441 Eastlake Avenue, Los Angeles, CA 90089, USA; 6 Clinical Medicine, Department of Medical Oncology, USC/Norris Comprehensive Cancer Center, 1441 Eastlake Avenue, Los Angeles, CA 90089, USA; 7 Department of Molecular Microbiology & Immunology and Urology, USC/Norris Comprehensive Cancer Center, 1450 Biggy Street, Los Angeles, CA 90033, USA; 8 USC/Norris Comprehensive Cancer Center, 1441 Eastlake Avenue, Los Angeles, CA 90089, USA; 9 Department of Pathology, City of Hope, 1500 East Duarte Road, Duarte, CA 91010, USA; 10 USC/Norris Comprehensive Cancer Center, 1450 Biggy Street, Los Angeles, CA 90033, USA; 11 Abbvie, 1000 Gateway Boulevard, South San Francisco, CA 94080, USA

**Keywords:** mCRPC, Radium 223, enzalutamide, STAT3, PEACE-3

## Abstract

Introduction: Prostate cancer has been generally resistant to immunotherapy approaches. Radiation can be immunostimulatory, but the extent to which standard prostate cancer treatments induce immune activation has not been well described. The bone-targeted radiopharmaceutical Radium223 (Ra223) has been proposed to enrich immune function, but clinical studies have not fully delineated whether this is true, or by what mechanisms. Enzalutamide has been shown to increase PD-L1 expression on dendritic cells, which could impact immune activation, though the extent to which this is associated with other evidence of immune activation remains uncertain, and combination strategies remain of interest. We performed a randomized phase II trial to evaluate whether Radium223 (Ra223) added to enzalutamide would induce greater immune activation and clinical responses compared to enzalutamide alone in men with metastatic castration-resistant prostate cancer (mCRPC). Methods: Eligible patients were randomized 2:1 to Arm A (enzalutamide 160 mg PO daily + Ra223 55 kBq/kg IV q4 weeks × 6 doses) or Arm B (enzalutamide 160 mg PO daily). Blood was collected at treatment start and during treatment to measure soluble immune checkpoint biomarkers (BTLA, TIM3, HVEM, GITR, LAG3, PD-1, CTLA-4, PD-L1, PD-L2, ICOS). Immunophenotyping by mass cytometry time of flight (CyTOF) was performed to measure peripheral blood mononuclear cell populations before and after treatment. CyTOF was used to determine changes in circulating immune cell population subsets before and after treatment. Biopsies were performed of an active bone metastatic lesion prior to study treatment and after at least 3 months. IHC was subsequently performed to examine changes in immune cell population subsets before and after treatment, and changes in pSTAT3 levels. Results: In total, 30 patients were enrolled, with median age 68. The median duration of follow up was 36 months. PSA responses, PFS, and OS were not significantly different between the two arms; however, the study was not powered for clinical endpoints. Peripheral blood and bone biopsy specimens were analyzed for immune correlatives. Soluble receptor concentrations showed significantly increased expression of PDL-2 in the combination arm, but this was not seen on CyTOF. Otherwise, there were no significant differences in markers of immune activation/exhaustion or immune cell population subsets in the combination arm and enzalutamide monotherapy arm. IHC also did not show a significant difference in immune cell population subsets in bone biopsy specimens before and after treatment in both arms. However, treatment with the combination arm did show significantly increased levels of pSTAT3 (*p* = 0.04), which was not seen in the enzalutamide monotherapy arm. Conclusions: Our study showed an overall lack of evidence for immune activation or cytokine induction with the combination, which does not make a strong case for combinatorial immunotherapy approaches. However, the combination did induce higher levels of pSTAT3, which has been implicated in radio-resistance. Therefore, the addition of a STAT3 inhibitor to the combination may be of interest to improve efficacy.

## Simple Summary

In this study, we looked at patients with metastatic hormone-resistant prostate cancer, and wanted to see whether adding Radium-223 to enzalutamide would cause their immune system to be more active. We found that adding Radium-223 did not make their immune system more active, but it did increase levels of a protein that helps the cancer become resistant to radiation therapy. Our results will be helpful for future studies aimed at improving this combination regimen.

## 1. Introduction

Metastatic castration-resistant prostate cancer (mCRPC) remains a lethal disease, despite many therapeutic advances over the last decade. While cellular immunotherapy with sipuleucel-T was among the first immunotherapies to show survival advantage in solid tumors, the survival prolongation in mCRPC was noted without objective responses or the induction of durable remissions [1]. Unlike other solid tumors, mCRPC does not respond well to immune checkpoint inhibitors (ICI) targeting PD-1, PD-L1 and CTLA-4 [2,3], though there is interest in combining these agents with other therapies. In particular, enzalutamide upregulates PD-L1 in dendritic cells [4].

Ra223 is an intravenous alpha emitting calcium mimetic therapy approved for the treatment of metastatic castration-resistant prostate cancer (mCRPC) with symptomatic bone involvement. In the ALSYMPCA trial, Ra-223 was found to prolong the survival of patients with mCRPC, in addition to reducing or delaying symptomatic skeletal-related events [5]. Unlike previous bone-targeted radiopharmaceuticals such as samarium-153 and strontium-89, Ra223 induces less myelosuppression, which allows for repeated dosing. While Ra223 clearly impacts marrow function and could conceivably lead to immune suppression, there has been interest in combining it with immune therapy, since radiation (including alpha particles) can induce cancer cell death in a way that is immunogenic [6,7]. In addition, there have been preclinical models suggesting that Ra223 can induce checkpoint inhibitor expression in the mCRPC bone microenvironment, potentially turning the transition from the traditionally “cold” microenvironment to a “hot” one [8]. However, some clinical evidence suggests a lack of change in CD8 cell numbers and subsets after a single dose of Ra223 [9]. Further correlative data are needed to better understand to what extent, and how, Ra223 yields immune activation.

Since soft tissue metastases are not the target of Ra223, combination therapy with more broadly acting therapies, i.e., androgen-receptor pathway inhibitors (ARPi) such as abiraterone or enzalutamide, is clinically appealing. Hormones, including androgens, have long been thought to play an important role in immune system function. For example, studies on prostate cancer patients undergoing androgen deprivation have shown an increased number of circulating pro-inflammatory T_H_1 T cells and a decreased number of immune-suppressing CD4+ Treg cells [10,11,12]. Using androgen-targeted agents in combination with Ra223 may provide a synergistic effect on immune activation.

Ra223 has already been studied in several combinations with androgen-targeting agents (e.g., abiraterone in the ERA-223 trial and enzalutamide in the PEACE-3 trial). No synergy was noted in the ERA-223 trial in terms of symptomatic skeletal event-free survival [13], but significant improvement in survival was seen in PEACE-3 [14]. However, there have been limited efforts to systematically evaluate immune activation induced by these agents alone or in combination.

We designed an open-label phase II study exploring the combination of Ra223 and enzalutamide in men with metastatic castration-resistant prostate cancer (mCRPC) with the aim of specifically evaluating whether the combination would induce greater immune activation, based on both circulating and tissue assessments, compared to enzalutamide alone. In addition, clinical outcomes were considered.

## 2. Methods

Approval was obtained from the institutional review boards at two independent collaborating cancer centers, the University of Southern California and City of Hope. All patients signed written informed consent. Trial Registration Number: NCT03344211.

The trial was designed with a 2:1 randomization between the experimental arm of enzalutamide 160 mg PO daily plus radium-223 55 kBq/kg IV q4 weeks × 6 doses and the control arm of enzalutamide alone, which was dosed in 4-week cycles. Eligible patients had histologically confirmed prostate cancer and active bone metastases (at least one site that had not been previously irradiated), and had developed castration resistance. At the time the study was designed it was still common practice to treat with enzalutamide after progression on abiraterone, thus patients whose disease had previously progressed on abiraterone were eligible. Prior taxane chemotherapy for castration-resistant disease was not permitted, but taxane chemotherapy used up front in the metastatic hormone-sensitive state was allowed provided that at least 6 months had elapsed since the final dose was administered. Adequate marrow function was required, defined as absolute neutrophil count >1500, hemoglobin >9.5 and platelets >100. In order to enroll, patients consented to a pre-treatment biopsy of an active bone metastasis performed under CT guidance; this was repeated after 3 cycles of treatment. Bone-supportive agents, such as denosumab and zoledronic acid, were required to be started at least 1 month prior to enrollment, and were administered concurrently, as per standard of care, unless contraindicated. Bone biopsy tissue was processed in a standard manner but without acid decalcification and paraffin embedding.

The study was designed with the dual primary endpoints of assessing changes in cancer involvement in the bone samples by histopathology and immunohistochemistry plus immune activation, comparing pre- to post-treatment biopsy and blood samples. During the study it became clear that the bone samples did not provide adequate tissue to evaluate cancer infiltration quantitatively. Thus, we are focusing on the analyses of immune activation.

The primary endpoint of immune activation was to be measured as a change in circulating biomarkers from baseline (day of treatment initiation) to post-treatment (at the end of cycle 1) as well as a change in tissue myeloid-derived suppressor cell (MDSC) STAT3 levels and tumor infiltration by lymphocytes. Based on previous analyses of Ra223 and its effect on circulating immune cell population subsets [9], the study was designed to have 85% power in detecting a 2-fold difference in median change from baseline to post-treatment in MDSC levels of STAT3 expression and cytokine levels between the study arms with 36 subjects enrolled, using a 2-sided 0.05 level t-test after log transformation and assuming a coefficient of variation of 70% or less.

Additional measures of immune activation included Luminex assay to test Soluble receptor concentrations of immune cell activation/exhaustion. The following were measured using the MilliPlex Human Immuno-Oncology Checkpoint Protein Panel per manufacturer’s instructions (Millipore, Billerica, MA, USA) and log-transformed and compared using a Wilcoxon Rank Sum test to detect differences between the two arms: BTLA, TIM3, HVEM, GITR, LAG3, PD-1, CTLA-4, PD-L1, PD-L2 and ICOS. Immunophenotyping by mass cytometry time-of-flight (CyTOF) was used to measure differences in immune cell population subsets and markers of immune cell activation/exhaustion between treatment arms using Wilcoxon rank sum. IHC was also performed on bone biopsy samples to measure differences in immune cell population subsets, as was pSTAT3 expression.

### 2.1. Immunophenotyping by Mass Cytometry Time-of-Flight (CyTOF)

Whole blood was drawn by venipuncture into ACD-A-containing blood on day of treatment initiation and at the end of cycle 1, approximately 4 weeks later. Blood from each participating center was processed on the day of collection or shipped overnight and processed the next day by a central lab (USC Beckman Center for Immune Monitoring). Peripheral blood mononuclear cells (PBMCs) were isolated over a Ficoll–hypaque density gradient, cryopreserved, and stored in vapor phase liquid nitrogen prior to batch testing.

Cryopreserved PBMCs were thawed in 37 °C prewarmed RPMI-1640 cell culture medium containing 10% FBS and washed. Cells were treated with 0.1 mg/mL DNase for 15 min to prevent downstream clumping, then washed with medium again. Cell count and viability was assessed with a Countess II (Thermo Fisher, Waltham, MA, USA). Three million cells per sample were stained with 1 mL of 1x Cell-ID Cisplatin (Standard Biotools, South San Francisco, CA, USA) for 5 min and washed 3 times afterwards with Maxpar^®^ Cell Staining buffer (Standard Biotools). Cells were resuspended in 40 µL of M Maxpar^®^ Cell Staining buffer and blocked with 10 µL of FcR Blocking Reagent (Miltenyi Biotec, Bergisch Gladbach, Germany) for 10 min. Then, 50 μL of 2x antibody cocktail consisting of ready-to-use Maxpar^®^ Human T-Cell Phenotyping Panel Kit combined with Human T Cell Immuno-Oncology Expansion Panel Kit (Standard Biotools) was added to each sample, and incubated for 30 min at room temperature. After incubation, cells were washed twice with Maxpar^®^ Cell Staining buffer. Cells were then fixed in 1 mL 1.6% paraformaldehyde for 10 min and spun to a pellet at 800× *g* for 5 min. Fixative was removed and the cell pellet was resuspended in 1 mL of the 125 nM Cell-ID Intercalator-Ir (Standard Biotools) and incubated overnight at 4 °C. For data acquisition, stained PBMCs were washed twice in Maxpar^®^ cell-staining buffer, and then washed twice in Maxpar^®^ Cell Acquisition Solution (Standard Biotools). Cells were then resuspended to a concentration of 1 × 10^6^ cells/mL in Maxpar^®^ Cell Acquisition Solution containing 0.1× EQ™ Four Element Calibration Beads. Each sample was acquired on the CyTOF Helios instrument (Standard Biotools) using CyTOF Software v9.1 (Standard Biotools). Longitudinal samples from a single patient were all stained and data were acquired in the same technical run to minimize variation. All acquisition data were normalized with CyTOF Software. Details on the CyTOF panel are displayed in Supplemental Table S1.

Supervised gating for cell populations was performed manually by a scientist without reference to clinical outcomes using CellEngine cloud-based flow cytometry analysis software (CellCarta, Montreal, QC, Canada). High-level cell population gates were tailored for each patient. Single marker gates were drawn uniformly across patients and timepoints. Immune populations were defined as in Supplemental Table S2. Viable cells were included in the analysis. Major cell subsets (total T cells, CD8+ T cells, CD4 Th cells, CD4 Tregs, and monocytes) were quantified as a percentage of the total leukocyte (CD45+) populations. Individual T cell activation or exhaustion markers were quantified as a percentage of cells expressing a particular marker within each parent population (CD8 T cells, CD4 Th cells, CD4 Tregs). An example of the gating strategy is shown in Supplemental Figure S1. Statistical tests for mass cytometry data used a 0.05 two-sided significance level, using GraphPad Prism software ver.10.2.2 (Boston, MA, USA). The statistical tests used are described in each figure legend.

### 2.2. Immunohistochemistry (IHC)

Phospho-Stat3 (Tyr705) IHC was performed on the Ventana Discovery Ultra IHC automated stainer (Ventana Medical Systems, Roche Diagnostics, Indianapolis, IN, USA).

Briefly, the tissue slides were deparaffinized, rehydrated and incubated with endogenous peroxidase activity inhibitor and antigen retrieval solution. Then the anti-Phospho-Stat3 (Tyr705) Rabbit monoclonal antibody (CLONE: D3A7, cat#: 9145, Cell Signaling Technology, Danvers, MA, USA) was incubated, followed by DISCOVERY anti-Rabbit HQ and DISCOVERY anti-HQ-HRP incubation. The stains were visualized with DISCOVERY ChromoMap DAB Kit, counterstained with hematoxylin (Ventana) and cover slipped. IHC whole-slide images were acquired with NanoZoomer S360 Digital Slide Scanner (Hamamatsu, Shizuoka, Japan) and viewed by NDP.view image viewer software 2.9.29. The intensity of the staining is noted in cells and the ratio of the weighted sum of the number of positive cells to the total number of detected cells is quantified as an H-score. The H-score was quantified as H-score = (1 × % of 1+ cells) + (2 × % of 2+ cells) + (3 × % of cells 3+).

The following are the immunostains with antibodies used to highlight the tumor and the different subsets of associated tumor infiltrating lymphocytes (TIL). The IHC was performed on a BOND-III automated stainer (Leica Biosystems, Nussloch, Germany). NKX3.1 (1:4000 ABCAM 186413), CD45 (LCA, RTU Leica PA0042), CD3 (RTU Leica PA0122), CD4 (CM 1:20 Cell Marque 104R-16), CD8 (RTU Leica PA0183), CD68 (RTU Leica PA0273) and PD-L1 (28-8) (1:100 Abcam ab205921). TILs were scored as follows: 0 = no positive lymphocytes; 1= 1–5 positive lymphocytes 2 = 5–10 positive lymphocytes; 3 = 11–20 positive lymphocytes; 4 = >21 positive lymphocytes. PD-L1 was considered positive when there was more than one positive staining tumor cell and negative if no tumor cells were highlighted.

## 3. Results

**Figure 1:**
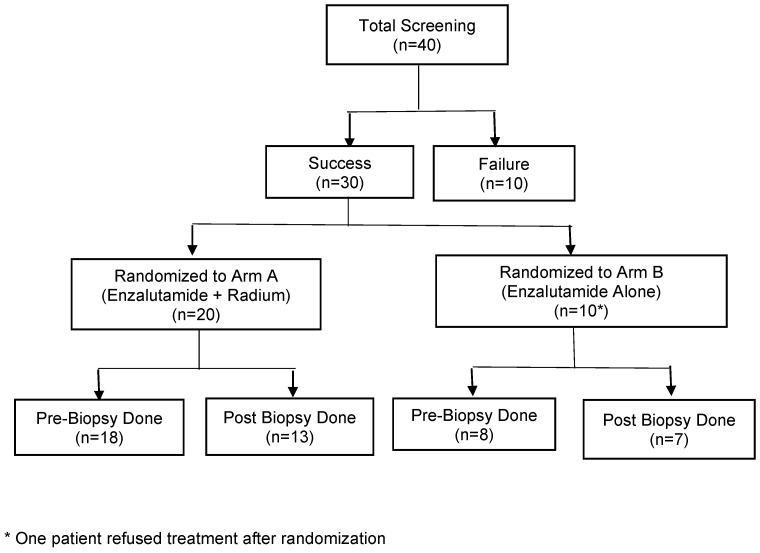
CONSORT diagram.

A CONSORT diagram (Figure 1) describes study accrual progress; enrollment was stopped early due to slowing accrual related to changes in the treatment landscape for mCRPC. A total of 29 patients were enrolled in the study: 20 patients on Arm A and 9 patients on Arm B. A summary of their baseline characteristics including demographics as well as notable clinical characteristics such as prior treatments can be seen in Table 1.

**Figure 2:**
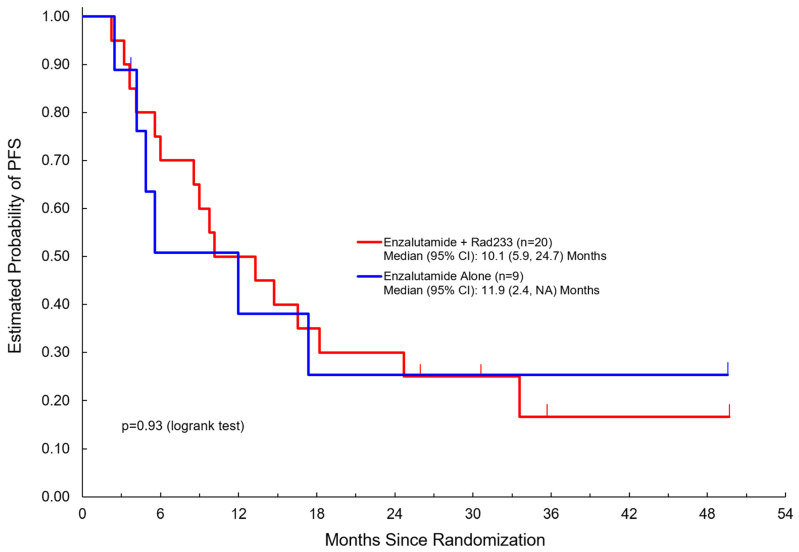
Progression-free survival among patients treated with enzalutamide + Radium223 compared to enzalutamide alone.

**Figure 3:**
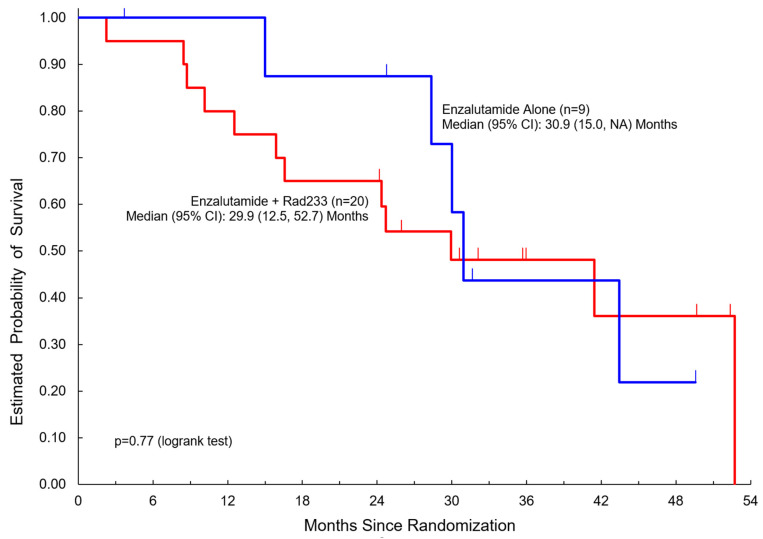
Overall survival among patients treated with enzalutamide + Radium223 compared to enzalutamide alone.

Table 2 summarizes clinical outcomes of the study. Median duration of follow-up was 36 months in both arms (3.7–52.4 months). Here, 19/20 patients in Arm A completed three or more cycles compared to 9/9 patients in Arm B. Notably, PSA response was not significantly different between the two arms. Disease control rate (DCR) was 75% in Arm A versus 67% in Arm B. There was no significant difference noted in PFS (median 10.1 months in Arm A versus 11.9 months in Arm B) or overall survival (median 29.9 months in Arm A versus 30.9 months in Arm B) (Figure 2 and Figure 3).

A summary of toxicities is listed in Table 3. The most common adverse events were fatigue and vascular/metabolic adverse events such as hot flashes, hypertension, or electrolyte abnormalities. As expected, hematologic adverse events including myelosuppression were seen only in Arm A with Ra223. Notably, no grade 3+ treatment-related adverse events were observed, and no fracture or skeletal-related events were noted during our study.

**Figure 4:**
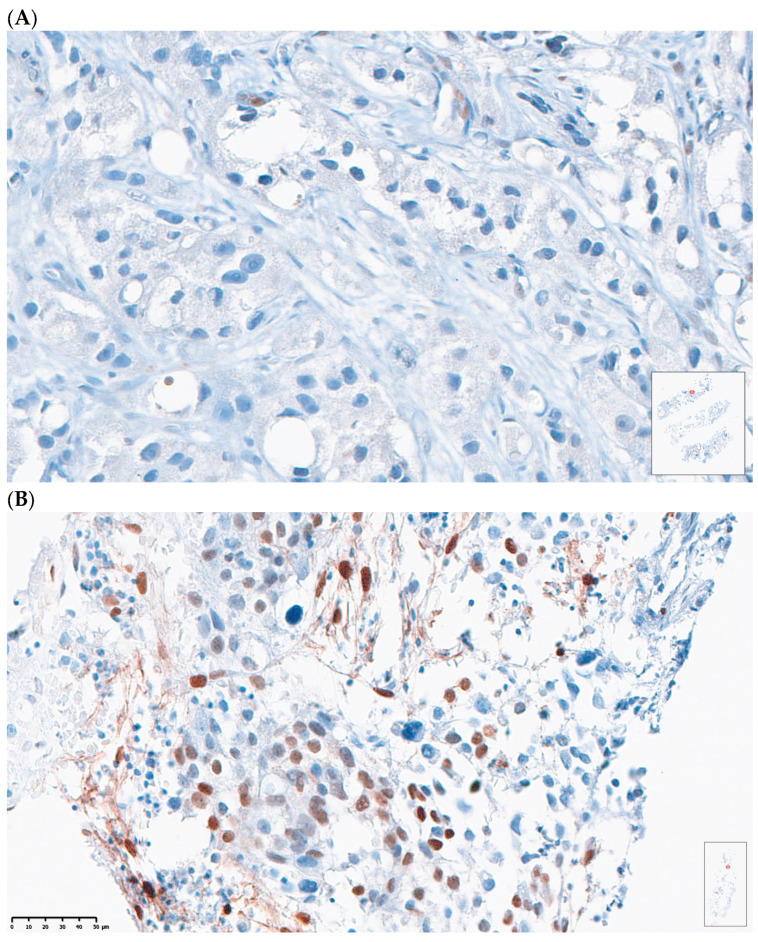
Representative case of (**A**) patient with low pSTAT3 expression and low H-score versus (**B**) patient with high pSTAT3 expression and high H-score.

**Figure 5:**
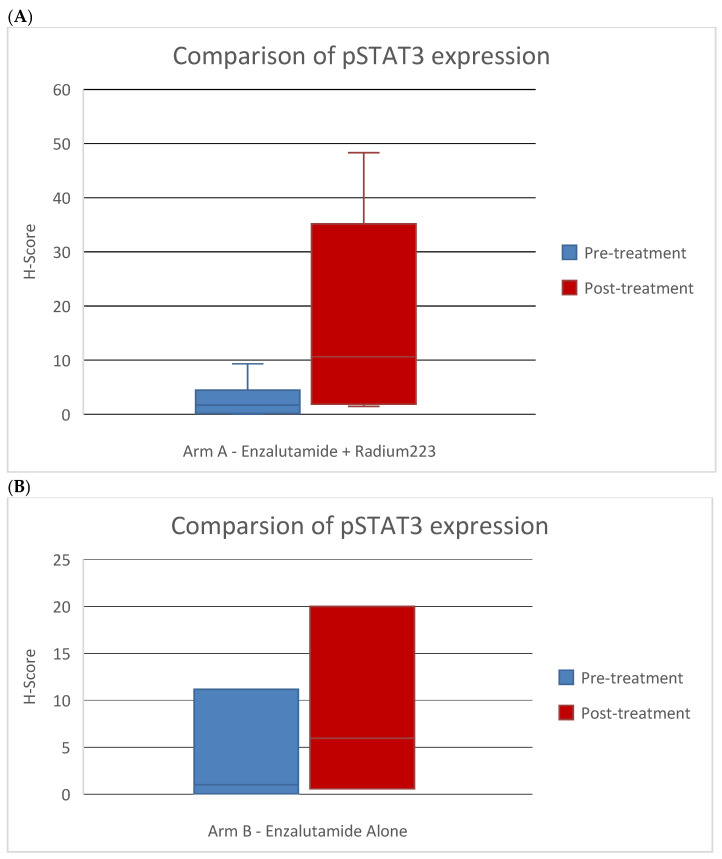
Comparison of pSTAT3 marker expression by IHC scoring in pre- and post-treatment bone biopsies in patients treated with (**A**) enzalutamide + Ra223 vs. (**B**) enzalutamide alone.

Pre-treatment bone biopsies were obtained in 18 patients in Arm A and 8 patients in Arm B on day of treatment initiation. Post-treatment biopsies were obtained in eight patients in Arm A and seven patients in Arm B on C4D1. IHC staining was performed to examine immune cell population subsets in pre- and post-treatment biopsies and pSTAT3 levels. An example of an IHC-stained specimen can be seen in Figure 4. The quantification of IHC staining for different immune cell population subsets showed no significant difference pre- and post-treatment (Supplementary Table S3). The quantification of pSTAT3 IHC staining in the combination arm showed significantly higher pSTAT3 levels after treatment (*p* = 0.04). An increase in pSTAT3 levels was also seen in the enzalutamide monotherapy arm, but this did not reach statistical significance (*p* = 0.6) (Figure 5).

**Figure 6:**
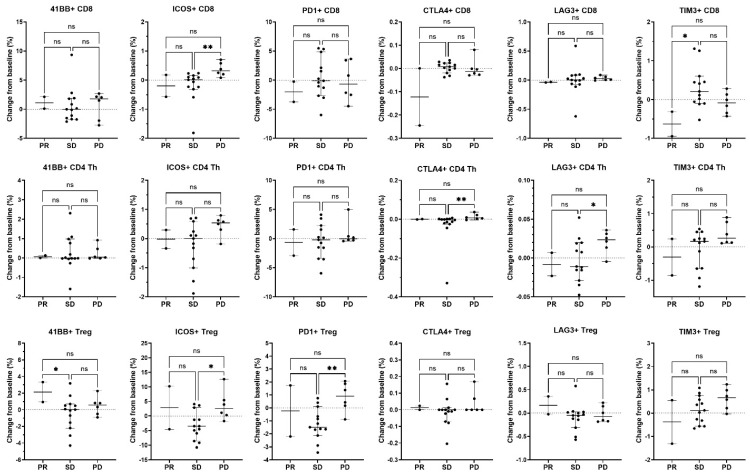
Association of changes in T cell activation and inhibitory molecules expressed on T cell subsets with best overall response. On-treatment changes from baseline in immune parameters expressed on CD8 T cells (top row), CD4 Th cells (middle row), and CD4 Tregs (bottom row) were related to the best overall clinical response in evaluable patients (n = 2 PR, n = 13 SD, and n = 6 PD) irrespective of treatment arm. For the activation or inhibition of molecule expression, the percent of parent population change was calculated as the frequency of marker-positive cells at baseline subtracted from the frequency of marker-positive cells at the on-treatment timepoint (end of cycle 1/beginning of cycle 2). Only patients who had both a baseline and an on-treatment longitudinal PBMC sample collected were included in this analysis. Individual data points are shown with horizontal lines representing median and with 95% CI. * *p* < 0.05, ** *p* < 0.01, ns, non-significant (nonparametric ANOVA and Kruskal–Wallis test for multiple comparisons).

**Figure 7:**
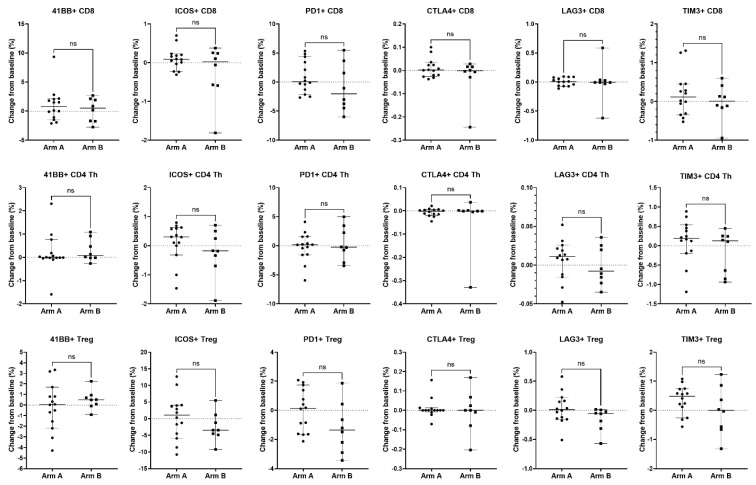
Association of changes in T cell activation and inhibitory molecules expressed on T cell subsets with treatment arm. Changes from baseline in immune parameters expressed on CD8 T cells (top row), CD4 Th cells (middle row), and CD4 Tregs (bottom row) were analyzed between patients treated with enzalutamide plus Radium-223 (Arm A, n = 14) or enzalutamide alone (Arm B, n = 8). For activation or inhibition of molecule expression, the percent of parent population change was calculated as the frequency of marker-positive cells at baseline subtracted from the frequency of marker-positive cells at the on-treatment timepoint (end of cycle 1/beginning of cycle 2). Only patients who had both a baseline and on-treatment longitudinal PBMC sample collected were included in this analysis. Individual data points are shown with horizontal lines representing median with 95% CI. ns, non-significant (nonparametric unpaired Mann–Whitney test).

**Figure 8:**
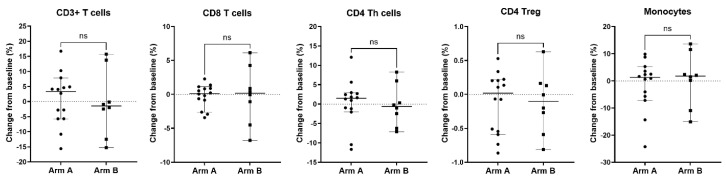
Change from baseline in circulating T cell subsets and monocyte population in patients treated with enzalutamide plus Radium-223 or enzalutamide alone. Percentages of CD3+ T cells, CD8+ T cells, CD4 Th cells, CD4 Tregs, and monocytes were determined based on total CD45+ PBMC from patients with longitudinal baseline and on-treatment samples from Arm A (enzalutamide plus Radium-223, n = 14) or Arm B (enzalutamide alone, n = 8). The percent change from baseline is shown for each population. Only patients who had both a baseline and on-treatment longitudinal PBMC sample collected were included in this analysis. Individual data points are shown with horizontal lines representing median with 95% CI. ns, non-significant (nonparametric unpaired Mann–Whitney test).

Peripheral blood was also analyzed for immune correlatives. Cytokines remained generally low at baseline and post-treatment time points. Soluble receptor concentrations of immune cell activation/exhaustion markers showed a notable increase in soluble PD-L2 levels in the combination arm after treatment (*p* = 0.0026) (Table 4), whereas others were not significantly changed. Immunophenotyping by CyTOF was also performed to examine differences in immune cell population subsets and immune cell activation/exhaustion markers before and after treatment. There was no difference detected in immune cell population subsets and immune cell activation/exhaustion markers (Figure 6, Figure 7 and Figure 8, Supplementary Figure S2).

## 4. Discussion

Radiation has been found to kill cancer cells in an immunogenic fashion [15], raising interest in combining radiotherapy with immunotherapy, though differential impacts of systemic radioactive therapy compared to external beam radiation techniques have not been completely delineated. Specifically, the immunologic impact of Ra223 has not been fully described, particularly in the bone tumor microenvironment (TME). Given that a previous study had noted the augmented expression of immune checkpoint proteins after Ra223 [8], we were specifically interested in changes in tissue markers of immune activation after Ra223, but given the established limitations of studying bone metastatic biopsy samples, we planned to look at activation status in circulating immune cell subsets as well. Overall, in our study, there was no strong evidence of immune activation induced by either enzalutamide or enzalutamide in combination with Ra223. Soluble receptor concentration measurements showed upregulation in PD-L2, but this was not seen on immunophenotyping by CyTOF, which is arguably more accurate. This fits with the limited prior literature, in which combining Ra223 has not been shown to consistently augment immune activation; for instance, combining Ra223 with sipuleucel-T did not increase antigen specific T cell activation as measured by ELISPOT [16], though there was some apparent clinical synergy. Our data extend the findings from a study of 15 men treated with Ra223 in which no increase in CTLA4 or PD-1 expression on CD8 cells was noted after a single dose [9], but changes in PD-L2 were seen after multiple doses and in the context of treatment with enzalutamide.

Of note, our study did find increased pSTAT3 levels in post-treatment biopsies in the combination arm. STAT3 is a promoter of MDSC immune regulatory activity and can be induced by radiation, along with PD-1, based on studies in head and neck cancer [17]. STAT3 promotes the immunosuppressive functions of MDSCs, such as the expression of IL-23 and ARG1 [18]. While this is a small study and the difference detected in this patient population can only be hypothesis-generating, it is of interest since STAT3 can be a contributor to resistance to radiation therapy [19]. In addition, the STAT3 signaling axis has been implicated in enzalutamide resistance [20]. The targeting of the STAT3 axis has been shown to reverse enzalutamide resistance in prostate cancer [21,22]. Therefore, the addition of a STAT3 inhibitor may be of interest to improve the efficacy of the combination. Several STAT3 inhibitors are undergoing early phase clinical testing (ex: AZD9150 NCT03421353, WP1066 NCT01904123 and TTI-101 NCT03195699).

In addition to the PD1/PDL1 axis, we also examined multiple immune checkpoints including ICOS. ICOS is an immune checkpoint protein that plays an important role in the tumor microenvironment of prostate cancer. In prostate cancer, ICOS has been found on tumor-infilitrating lymphocytes including Tregs, and high expression induces an immunosuppressive environment. The inhibition of these ICOS+ Tregs has been shown to greatly enhance antitumor responses [23]. In addition, the combination of ICOS blockade with the inhibition of other checkpoints, such as CTLA-4, seems to be particularly effective in generating anti-tumor responses in prostate preclinical models [24]. Ultimately, in our samples, there was no evidence of ICOS upregulation, but there was evidence of PDL2 upregulation in patients treated with the combination of enzalutamide plus Radium223, determined by luminex but not by flow cytometry. This is likely due to the limitations of luminex as a measure for immunophenotyping. Nonetheless, PDL2 has been shown to be important in immune regulation in prostate cancer, based on a series of over 9000 prostatectomy tissue samples [25]. Higher expression was associated with an increased risk of biochemical recurrence, but also with response to salvage radiation therapy. The further exploration of this pathway in advanced prostate cancer may be warranted, as expression levels and impact on cancer control could change over time.

Studying immune activation only in the circulation could lead one to miss important effects in the TME. Thus, we performed matched biopsies before and after treatment to better evaluate the baseline and changes in tumor immune infiltration. Unfortunately, the quality of biopsy specimens was variable, which reduced the power of the analysis. This has been a limiting factor in other attempts to interrogate cancer and biology and treatment changes in bone metastases; for instance, Armstrong and colleagues performed pharmacokinetic analyses of Ra223 using bone biopsy before and after treatment, but only 5 of 20 enrolled subjects had adequate matched tissue specimens [26]. Perhaps rapid postmortem tissue collection will be needed to offer better studies of clinical DNA, RNA, and protein changes in bone metastases, though one group utilized fresh frozen tissue from patients undergoing the surgical resection of prostate metastatic tissue due to spinal cord compression [27].

Regarding clinical outcomes, the final analysis did not show a significant difference in the two arms in terms of PSA response, PFS, and OS. However, our study was not powered to detect differences in these clinical endpoints. More recently, outcomes of PEACE-3 examining the same combination of Ra223 and enzalutamide showed a significant rPFS and OS benefit in the combination [14]. With regard to safety, our study showed that the combination was tolerable. Of particular note, we did not note any fractures or skeletal-related events. However, the interim safety report from PEACE-3, which was reported at a much later date after our study, showed increased fracture risk with the combination [28]. Our study showed no fracture events, which may be explained by institutional policy, whereby most patients are on some form of bone protecting agents (BPA). Baseline BPA was not recorded given the timing of our study relative to the interim safety report from PEACE-3. Additionally, given that this study preceded the introduction of PARP inhibitors in mCRPC, the somatic and germline analysis of patients was not commonly performed and therefore was not included in this study, though it would be of interest in future studies.

## 5. Conclusions

Overall, we showed that the combination of enzalutamide and Ra223 did not increase immune activation compared to enzalutamide alone. Recent data from PEACE-3 show improved clinical outcomes with the combination, and our results suggest that this is likely attributed to non-immune related mechanisms. pSTAT3 levels were significantly increased with the combination, and the addition of a STAT3 inhibitor may be a combination of interest for future clinical study.

## Figures and Tables

**Table 1:** Demographics and clinical baseline characteristics.

	Total Patients	Arm A Enzalutamide + Rad233	Arm B Enzalutamide
**Total Patient Randomized and Treated**	29 *	20	9 *
**Age**			
Median (Range)	68 (53–87)	67 (57–87)	68 (53–78)
**Race/Ethnicity**			
White	19 (66%)	13 (65%)	6 (67%)
Hispanic	6 (21%)	3 (15%)	3 (33%)
Black or African American	2 (7%)	2 (10%)	0
Asian	2 (7%)	2 (10%)	0
**ECOG Performance Status**			
0	27 (93%)	18 (90%)	9 (100%)
1	2 (7%)	2 (10%)	0
**Gleason Score**			
7	6 (24%)	4 (25%)	2 (22%)
>7	19 (76%)	12 (75%)	7 (78%)
Median (Range)	8 (7–10)	8.5 (7–10)	8 (7–9)
Unknown	4	4	
Visceral Mets Involved at Study Entry			
No	16 (62%)	12 (67%)	4 (50%)
Yes	10 (38%)	6 (33%)	4 (50%)
Unknown	3	2	1
**Received Prior Chemotherapy**			
No	15 (52%)	9 (45%)	6 (67%)
Yes	14 (48%)	11 (55%)	3 (33%)
**Baseline Testosterone Level**			
Median (Range)	10.9 (2.5–65.5)	11.0 (2.5–65.5)	10.0 (7.0–22.0)
Missing	5	3	2
**Baseline PSA Level**			
Median (Range)	13.3 (0.5–1760)	12.3 (0.5–1760)	30.5 (2.7–142.1)
**Disease Involvement**			
Bone	29 (100%)	20 (100%)	9 (100%)
Non-regional Lymph Nodes	5 (17%)	3 (15%)	2 (22%)
Pelvis	3 (10%)	2 (10%)	1 (11%)
Visceral	4 (13%)	2 (10%)	2 (22%)

* One patient who withdrew from study prior to treatment after randomization was excluded.

**Table 2:** Study outcomes.

	Number Patients			
	Total Patients (n = 29)	Arm A Enzalutamide + Rad233 (n = 20)	Arm B Enzalutamide (n = 9)	*p*-Value
**Number of Cycles Given**				
1 or 2 Cycles	1 (3%)	1 (5%)	0	
≥3 Cycles	28 (97%)	19 (95%)	9 (100%)	
Median (Range)	6 (1–6)	6 (1–6)	6 (4–6)	
**PSA Maximum Changed from Baseline**				0.30 ^
Median (Range)	−57% (−100–62%)	−53% (−100–62%)	−61% (−99–−15%)	
**PSA Changed from Baseline at 12 Weeks since Treatment Start**				0.30 ^
Median (Range)	−39% (−100–140%)	−28% (−100–140%)	−61% (−97–79%)	
Not available		2		
**PSA30 Response**				0.11 *
No	10 (34%)	9 (45%)	1 (11%)	
Yes	19 (66%)	11 (55%)	8 (89%)	
**Best Clinical Overall Response**				0.56 *
PR	2 (7%)	1 (5%)	1 (11%)	
SD	19 (62%)	14 (70%)	5 (56%)	
PD	8 (28%)	5 (25%)	3 (33%)	
**Off Treatment Reason**				
Complete Treatment	21 (72%)	16 (80%)	5 (56%)	
Disease Progression	7 (24%)	4 (20%)	3 (33%)	
Unacceptable Toxicity	1 (3%) ^#^	0	1 (11%)	
**Overall Survival**				
Median (95% CI)	30.9 (24.3, 52.7) Mo.	29.9 (12.5, 52.7) Mo.	30.9 (15.0, NA) Mo.	0.77 ~
**Progression Free Survival**				
Median (95% CI)	11.9 (5.5, 17.4) Mo.	10.1 (5.9, 24.7) Mo.	11.9 (2.4, NA) Mo.	0.93 ~
**Duration of Follow-Up**				
Median (Range)	36.0 (3.7–52.4) Mo.	36.0 (24.2–52.4) Mo.	49.6 (3.7–49.6) Mo.	

^ *p*-value based on Wilcoxon test; * *p*-value based on Fisher’s exact test; ^#^ Grade 2 pain attribution to biopsy; ~ *p*-value based on logrank test.

**Table 3:** Summary of toxicities during treatment.

	Arm A: Radium223 + Enzalutamide (n = 20)			Arm B: Enzalutamide (n = 9)		
Treatment-Related Adverse Event (TRAE)	Any Grade TRAE (%)	Grade 1	Grade 2	Any Grade TRAE (%)	Grade 1	Grade 2
Leukopenia	6 (30%)	4 (20%)	2 (10%)	0 (0%)	0 (0%)	0 (0%)
Hyponatremia	6 (30%)	6 (30%)	0 (0%)	1 (11%)	1 (11%)	0 (0%)
Neutropenia	5 (25%)	2 (10%)	3 (15%)	0 (0%)	0 (0%)	0 (0%)
Fatigue	5 (25%)	4 (20%)	1 (5%)	4 (44%)	4 (44%)	0 (0%)
Anemia	3 (15%)	3 (15%)	0 (0%)	0 (0%)	0 (0%)	0 (0%)
Hot Flashes	3 (15%)	3 (15%)	0 (0%)	1 (11%)	1 (11%)	0 (0%)
Nausea	3 (15%)	3 (15%)	0 (0%)	1 (11%)	0 (0%)	1 (11%)
Dizziness	3 (15%)	3 (15%)	0 (0%)	3 (33%)	2 (22%)	1 (11%)
Thrombocytopenia	2 (10%)	2 (10%)	0 (0%)	0 (0%)	0 (0%)	0 (0%)
Diarrhea	2 (10%)	2 (10%)	0 (0%)	0 (0%)	0 (0%)	0 (0%)
Myalgias	2 (10%)	2 (10%)	0 (0%)	1 (11%)	1 (11%)	0 (0%)
Constipation	1 (5%)	0 (0%)	1 (5%)	1 (11%)	1 (11%)	0 (0%)
Elevated Alkaline Phosphatase	1 (5%)	1 (5%)	0 (0%)	1 (11%)	1 (11%)	0 (0%)
Hyperglycemia	1 (5%)	1 (5%)	0 (0%)	2 (22%)	2 (22%)	0 (0%)
Dysgeusia	1 (5%)	1 (5%)	0 (0%)	0 (0%)	0 (0%)	0 (0%)
Hypertension	1 (5%)	0 (0%)	1 (5%)	3 (33%)	0 (0%)	3 (33%)

There were no grade 3 or 4 toxicities observed.

**Table 4:** Comparison of soluble protein levels of immune checkpoint inhibitors before and after treatment.

Immune Analyte	Arm A Enzalutamide + Rad233		Arm B Enzalutamide	
	Mean Expression (End of Treatment vs. Baseline in pg/mL)	*p*-Value	Mean Expression (End of Treatment vs. Baseline in pg/mL)	*p*-Value
PDL-2	17,817 vs. 16474	***p* = 0.0026**	1787 vs. 1641	*p* = 0.57
BTLA	2246 vs. 2063	*p* = 0.57	1787 vs. 1641	*p* = 0.8
TIM-3	3233 vs. 3072	*p* = 0.57	3239 vs. 3350	*p* = 1
HVEM	2310 vs. 2163	*p* = 0.078	2868 vs. 2861	*p* = 1
LAG-3	115,977 vs. 112,772	*p* = 0.78	159,067 vs. 119,192	*p* = 0.23
GITRL	1068 vs. 1046	*p* = 0.42	759 vs. 711	*p* = 0.83
PD-1	1717 vs. 1690	*p* = 0.65	1450 vs. 1423	*p* = 1
CTLA-4	164 vs. 155	*p* = 0.87	119 vs. 102	*p* = 0.57
PDL-1	294 vs. 277	*p* = 0.91	202 vs. 102	*p* = 0.88
ICOS	3096 vs. 3116	*p* = 0.91	1773 vs. 1885	*p* = 0.88

## Data Availability

Data for the study can be made available following request from the corresponding author and with permission obtained by Bayer.

## Supplementary Materials

The following supporting information can be downloaded at: https://www.mdpi.com/article/10.3390/cancers17101730/s1, Table S1. Immune phenotyping CyTOF antibody panel; Table S2. Immune cell population definitions; Figure S1. CyTOF immunophenotyping gating strategy example; Figure S2. Example of TIL staining in biopsy specimens; Table S3. Comparison of TIL marker expression by IHC scoring in pre- and post-treatment bone biopsies in patients treated with enzalutamide + Ra223 (Arm A) vs. enzalutamide alone (Arm B); Figure S3. Circulating T cell subsets and monocyte populations in patients treated with enzalutamide plus Radium-223.
