# Supplementary material for: Immune Modulation During Treatment with Enzalutamide Alone or with Radium-223 in Patients with Castration Resistant Prostate Cancer
Source: Cancers (Basel). 2025 May 21;17(10):1730. doi: 10.3390/cancers17101730 (PMC12110403; doi:10.3390/cancers17101730)
Supplement: Supplementary file 1 [file cancers-17-01730-s001.zip › cancers-3585512-supplementary.pdf]

Supplemental Figures and Tables

Supplemental Table S1. Immune phenotyping CyTOF antibody panel

Target	Clone	Metal
CD11a	HI111	142Nd
CD4	RPA-T4	145Nd
CD8a	RPA-T8	146Nd
CD16	3G8	148Nd
CD25	2A3	149Sm
CD134/OX40	ACT35	150Nd
CD95/Fas	DX2	152Sm
CD366/TIM-3	F38-2E2	153Eu
CD45	HI30	154Sm
CD279/PD-1	EH12.2H7	155Gd
CCR7	G043H7	159Tb
CD152/CTLA-4	14D3	161Dy
CD69	FN50	162Dy
CD45RO	UCHL1	165Ho
CD44	BJ18	166Er
CD27	O323	167Er

CD178/ICOS	C398.4A	168Er
CD45RA	HI100	169Tm
CD3	UCHT1	170Er
CD57	HCD57	172Yb
CD127/4-1BB	4B4-1	173Yb
HLA-DR	L243	174Yb
CD223/LAG3	11C3C65	175Lu
CD127	A019D5	176Yb

Supplemental Table S2. Immune cell population definitions

**Population**	**Model Phenotype (cell surface markers)**
Total Leukocytes	CD45+
Monocyte	CD45+/CD3-/CD44+/CD11a+/HLA-DR+/CD45RA-/CD16-
CD16+ NK (natural killer cell)	CD45+/CD3-/HLA-DR-/CD57+/CD16+
T cell	Live/CD45+/CD3+
CD4 T cell	Live/CD45+/CD3+/CD4+
CD4 Treg	Live/CD45+/CD3+/CD4+/CD127-/CD25hi
CD4 Th (T helper)	Live/CD45+/CD3+/CD4+/CD127+/CD25low
CD4 T EM1 (Effector Memory 1)	CD4 Th cell + CD45RA-/CD27+/CCR7-
CD4 T EM2 (Effector Memory 2)	CD4 Th cell + CD45RA-/CD27-/CCR7+
CD4 T EM3 (Effector Memory 3)	CD4 Th cell + CD45RA-/CD27-/CCR7-
CD4 T CM (Central Memory)	CD4 Th cell + CD45RA-/CD27+/CCR7+

CD4 T EMRA (Effector Memory CD45RA-positive)	CD4 Th cell + CD45RA+/CD27-/CCR7-
CD4 T naïve	CD4 Th cell + CD45RA+/CD27+/CCR7+/CD95-
CD4 T SCM (Stem Cell Memory)	CD4 Th cell + CD45RA+/CD27+/CCR7+/CD95+
CD8 T cell	Live/CD45+/CD3+/CD8+
CD8 T EM1 (Effector Memory 1)	CD8 T cell + CD45RA-/CD27+/CCR7-
CD8 T EM2 (Effector Memory 2)	CD8 T cell + CD45RA-/CD27-/CCR7+
CD8 T EM3 (Effector Memory 3)	CD8 T cell + CD45RA-/CD27-/CCR7-
CD8 T CM (Central Memory)	CD8 T cell + CD45RA-/CD27+/CCR7+
CD8 T EMRA (Effector Memory CD45RA-positive)	CD8 T cell + CD45RA+/CD27-/CCR7-
CD8 T naïve	CD8 T cell + CD45RA+/CD27+/CCR7+/CD95-
CD8 T SCM (Stem Cell Memory)	CD8 T cell + CD45RA+/CD27+/CCR7+/CD95+

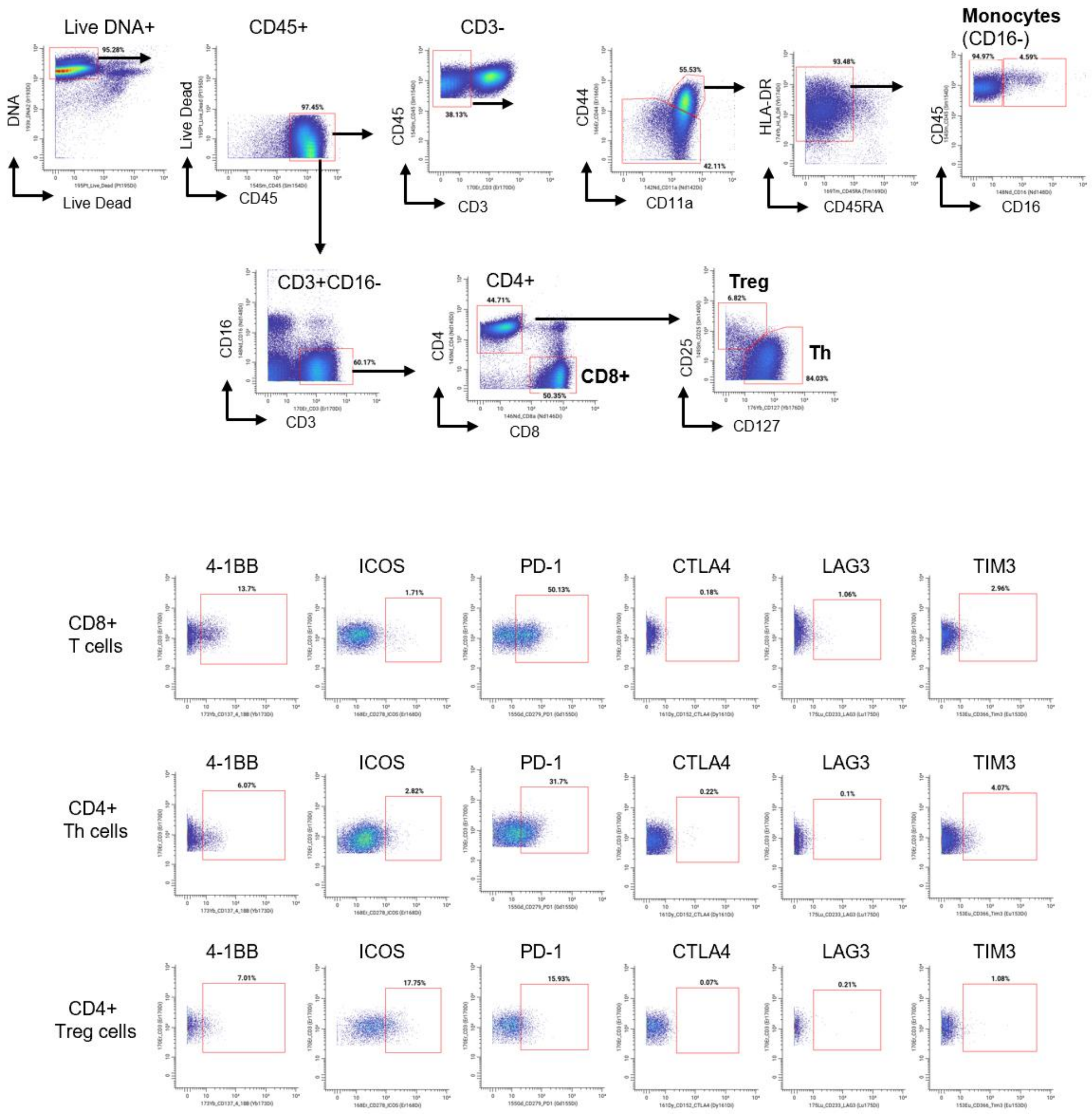

Supplemental Figure S1. CyTOF immunophenotyping gating strategy example.

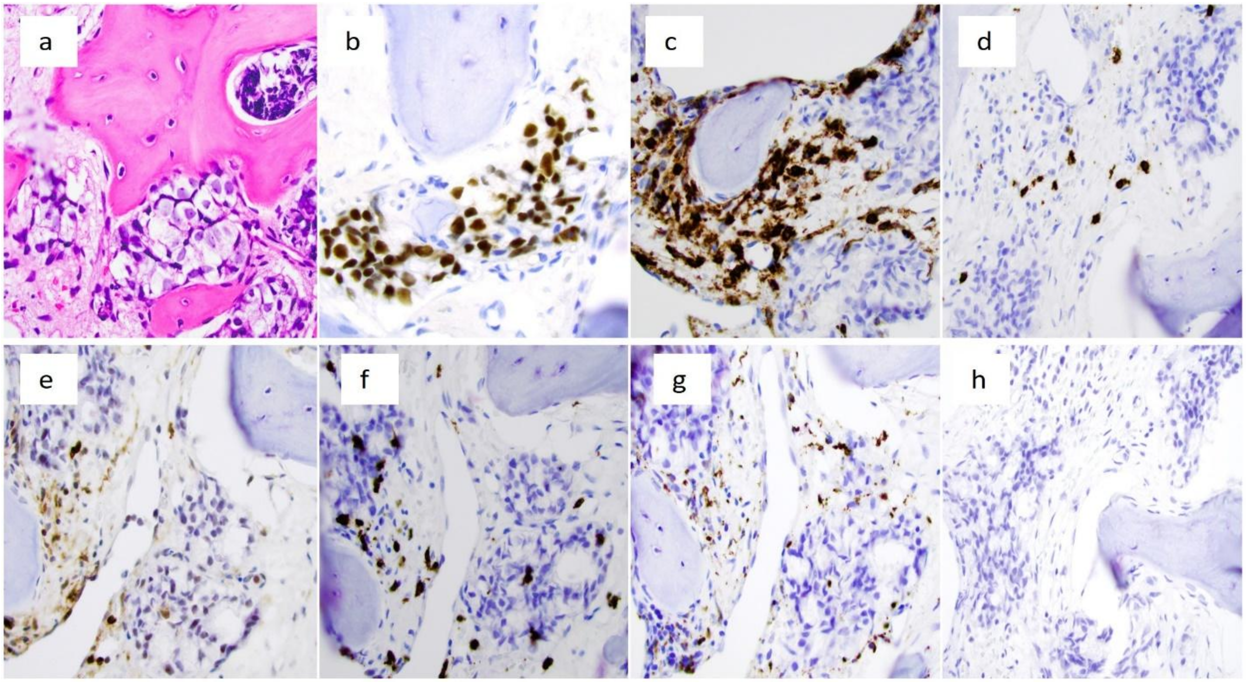

Supplemental Figure S2. Example of TIL staining in biopsy specimens. a. H/E with nests of prostate adenocarcinoma adjacent to bony trabeculae in this sample of a bony metastasis b. NKX 3.1 immunostain highlights tumor cells of prostatic origin c. CD45 immunostain highlights haematopoietic cells within the bone marrow d. CD3 highlights CD3 subset of T-cells e. CD4 highlights CD4 subset of T-cells f. CD8 highlights CD8 subset of T-cells g. CD68 highlights histiocytes h. PD-L1 is negative is tumor cells

Supplemental Table S3. Comparison of TIL marker expression by IHC scoring in pre- and post-treatment bone biopsies in patients treated with enzalutamide + Ra223 (Arm A) vs. enzalutamide alone (Arm B). IHC scoring defined as follows: 0 = no positive lymphocytes; 1= 1-5 positive lymphocytes 2 = 5-10 positive lymphocytes; 3 = 11-20 positive lymphocytes; 4 = > 21 positive. P values of degree in change in IHC scoring pre- and post-treatment between the two arms is reported.

	**Arm 1**	**Arm 2**	**p-value**
**CD45**			0.861
Mean Pre-treatment	2.27	2.25	
Mean Post-treatment	2.82	2.50	
**CD3**			0.65
Mean Pre-treatment	1.55	1.50	
Mean Post-treatment	1.82	1.50	
**CD4**			0.104
Mean Pre-treatment	1.36	1.25	
Mean Post-treatment	1.45	2.25	
**CD8**			0.604
Mean Pre-treatment	1.36	1.25	
Mean Post-treatment	1.45	1.50	
**CD68**			0.519
Mean Pre-treatment	2.36	1.75	
Mean Post-treatment	2.45	2.00	

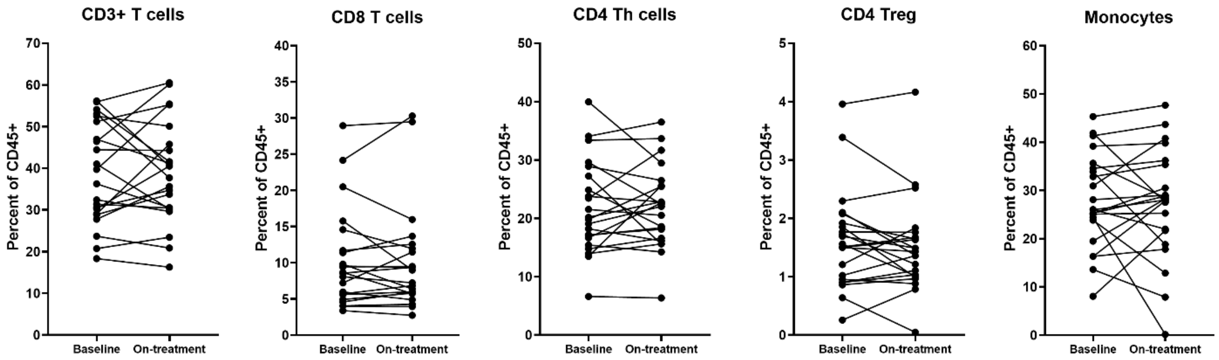

Supplemental Figure S3. Circulating T cell subsets and monocyte population in patients treated with enzalutamide plus Radium-223 (Arm A, n=14) and enzalutamide alone (Arm B, n=8). Percentage of CD3+ T cells, CD8+ T cells, CD4 Th cells, CD4 Tregs, and monocytes were determined gated on total CD45+ PBMC from patients with longitudinal baseline and on-treatment samples.
